# Comparison of Extended Skin Cancer Screening Using a Three-Step Advanced Imaging Programme vs. Standard-of-Care Examination in a High-Risk Melanoma Patient Cohort

**DOI:** 10.3390/cancers16122204

**Published:** 2024-06-12

**Authors:** Frank Friedrich Gellrich, Nadia Eberl, Julian Steininger, Friedegund Meier, Stefan Beissert, Sarah Hobelsberger

**Affiliations:** 1 Department of Dermatology, Faculty of Medicine and University Hospital Carl Gustav Carus, Technische Universität Dresden, 01307 Dresden, Germany; nadia.eberl@ukdd.de (N.E.); julian.steininger@ukdd.de (J.S.); friedegund.meier@ukdd.de (F.M.); stefan.beissert@ukdd.de (S.B.); sarah.hobelsberger@ukdd.de (S.H.); 2 Skin Cancer Center at the University Cancer Center, National Center for Tumor Diseases (NCT/UCC), 01307 Dresden, Germany

**Keywords:** melanoma, total body photography, reflectance confocal microscopy, digital dermoscopy, skin cancer screening

## Abstract

**Simple Summary:**

This study explored advanced diagnostic methods that are used for identifying skin cancer in high-risk melanoma patients. A total of 410 patients were examined using a combination of three advanced imaging techniques: 3D total body photography, digital dermoscopy, and reflectance confocal microscopy. These methods were used in addition to regular skin exams. Results showed that the specialized imaging detected 16 melanomas in 39 removed pigmented lesions, while regular exams detected only 7 melanomas in 163 removed lesions. This indicates that specialized imaging was much more efficient. Each imaging method found melanomas that the others did not. The study concludes that combining these three imaging techniques improves melanoma detection and reduces unnecessary skin removals in high-risk patients.

**Abstract:**

Modern diagnostic procedures, such as three-dimensional total body photography (3D-TBP), digital dermoscopy (DD), and reflectance confocal microscopy (RCM), can improve melanoma diagnosis, particularly in high-risk patients. This study assessed the benefits of combining these advanced imaging techniques in a three-step programme in managing high-risk patients. This study included 410 high-risk melanoma patients who underwent a specialised imaging consultation in addition to their regular skin examinations in outpatient care. At each visit, the patients underwent a 3D-TBP, a DD for suspicious findings, and an RCM for unclear DD findings. The histological findings of excisions initiated based on imaging consultation and outpatient care were compared. Imaging consultation detected sixteen confirmed melanomas (eight invasive and eight in situ) in 39 excised pigmented lesions. Outpatient care examination detected seven confirmed melanomas (one invasive and six in situ) in 163 excised melanocytic lesions. The number needed to excise (NNE) in the imaging consultation was significantly lower than that in the outpatient care (2.4 vs. 23.3). The NNE was 2.6 for DD and 2.3 for RCM. DD, 3D-TBP, or RCM detected melanomas that were not detected by the other imaging methods. The three-step imaging programme improves melanoma detection and reduces the number of unnecessary excisions in high-risk patients.

## 1. Introduction

Melanoma is a skin tumour with high metastasis and late-stage mortality rates [1,2,3]. Due to the steadily increasing incidence in recent decades, melanoma represents a significant health problem [4]. While its lymphogenous or distant metastasis stage requires complex and expensive tumour therapies [5] and regular staging examinations, the localised stage can be cured by radical excision [6]. Therefore, early detection is of great importance, especially in people at high risk of developing melanoma. Risk factors include a large number of congenital or atypical nevi [7,8,9,10]; a personal or family history of melanoma [11]; ultraviolet radiation, especially through high intermittent sun exposure [12,13], immunosuppression [14]; and genetic predisposition [15,16].

For some years, new diagnostic options have been used to improve skin cancer screening. Total body photography (TBP) can enhance the early detection of melanoma [17,18,19] and is therefore recommended for high-risk populations by the European interdisciplinary guideline for melanoma [2]. Three-dimensional TBP (3D-TBP) enables the three-dimensional imaging of almost the entire body surface. The position of each skin lesion on the body surface is saved with the image information, allowing automatic monitoring over time [20].

Melanoma detection by way of dermoscopy is better than through a naked eye examination, achieving a sensitivity of 79% and a specificity of 89%. It is therefore regularly used in dermatological skin examinations [21]. Sequential digital dermoscopy (DD) can further improve the detection of difficult-to-diagnose melanocytic lesions through side-by-side image comparisons [22,23,24] and is recommended for high-risk patients with a high nevus count [2]. The combined use of TBP and DD, the ‘two-stage digital follow-up method’ [25,26] frequently reported in the literature, allows for the early diagnosis of melanoma in high-risk patients [18].

Reflectance confocal microscopy (RCM) enables skin assessment to a depth of approximately 200 µm at a cellular resolution. Prospective studies have shown that RCM increases the diagnostic specificity of equivocal dermatoscopic findings [27,28,29,30].

The use of these modern diagnostic tools is limited due to their cost and time constraints, leading to their frequent unavailability in outpatient care. As these methods are recommended for high-risk patients [2], offering extended skin examinations at specialised centres seems reasonable. An imaging consultation was established in the dermatology department at Dresden University Hospital to deliver extended skin cancer screening. This study compared the extended examination to routine outpatient examinations.

## 2. Materials and Methods

This prospective single-centre study included 410 patients treated at the University Hospital Carl Gustav Carus, Dresden, between 21 April 2021 and 8 January 2024. The study protocol complied with the Declaration of Helsinki and was approved by the institutional ethics committee (Ethics Committee at TU Dresden, BO-EK-97022021). The patients gave written informed consent to collect, store, and analyse their personal and imaging data during the consultation.

Patients at high risk for developing melanoma were offered the opportunity to participate in an extended skin screening imaging consultation in addition to outpatient skin examinations. Alternating skin cancer screening in specialised centres and outpatient care for high-risk patients is a common practice in Germany. Patients with a history of melanoma, over 50 melanocytic nevi, dysplastic melanocytic nevi (diagnosed clinically and/or histologically), gene defects that increased the risk for developing melanoma, or immunosuppression were included. Depending on the dermatologist’s assessment in the imaging consultation, patients underwent follow-up examinations 3, 6, or 12 months later. The times of the outpatient consultations were not recorded and were not necessarily synchronised with the appointments in the imaging consultation. Many patients alternated between imaging consultations and outpatient care. Some patients were examined more frequently in one or the other consultation. In addition to analogue dermoscopy, the skin imaging consultation included DD, 3D-TBP, and RCM.

The patients underwent a physical examination and 3D-TBP during each imaging consultation visit, comparing the images with those from previous visits. New or changed and suspicious lesions in the clinical examination were examined dermoscopically. No additional diagnostic activity was performed if a lesion was deemed benign in dermoscopy. Otherwise, a DD was performed. Dermoscopically suspicious lesions were additionally examined with RCM. If RCM indicated that a lesion was benign with a high degree of certainty, a follow-up examination was recommended or no further diagnostic steps were taken. Unclear or malignant lesions in RCM underwent excision.

The histological findings were obtained by different pathologists in different laboratories. The pathologists received a written request. Dermoscopy photos or RCM images were not supplied. At least one immunohistochemical staining (Melan-A, SOX 10, PRAME, S-100, HMB-45) was used for the diagnosis of melanoma. Nevi and dysplastic nevi were partly diagnosed using H and E staining only. The diagnosis of invasive melanomas was confirmed by a second pathologist.

Indications for excisions in the imaging consultations and outpatient care examinations were made independently of each other. The histological findings of the two approaches were compared, and the influence of DD, RCM, and 3D-TBP on diagnosis was investigated.

We used VECTRA WB360s (Canfield Scientific Inc., Fairfield, NJ, USA) for 3D image acquisition, a VivaCam D200 (VivaScope GmbH, Munich, Germany) camera for DD, and a VivaScope 3000 (VivaScope GmbH) for RCM.

Statistical analysis was performed using IBM SPSS Statistics for Windows, Version 28.0 (IBM Corp., Armonk, NY, USA). Tests were two-sided, and statistical significance was set at *p* < 0.05. The chi-squared test and Fisher’s exact test explored intergroup diagnostic differences within and between approaches.

## 3. Results

Characteristics of the 410 patients who underwent extended skin cancer screening, 224 males and 186 females, are presented in Table 1. Thirteen patients ended the study prematurely due to death, relocation, own request, or missing appointments. A total of 1070 follow-up visits were carried out, 1–8 per patient, with an average of 2.1 (SD 1.1). The follow-up period was 0–33 months, with a mean of 14.9 (SD 8.5) months. The inter-visit interval was 1–24 months, with an average of 8.4 (SD 3.4) months.

The average patient age was 51.6 (range 18–84, SD 13.1) years. Of the 410 patients, 285 had a history of between 1 and 11 melanomas (mean 1.3, SD 1.0), 49 with in situ melanoma, and 236 with invasive melanoma. Fifty-one patients had a history of lymphogenous or distant metastases.

At study inclusion, 36 patients had fewer than 50 melanocytic nevi, 70 patients had 50–99 nevi, and 304 patients had more than 100 nevi. Of these, 257 patients had dysplastic nevi.

Among the patients, 7 had a genetic risk for developing melanoma and 16 were currently taking or had previously taken immunosuppressive medication (methotrexate, azathioprine, mycophenolic acid, TNF-alpha inhibitors, prednisolone, calcineurin inhibitors, and/or chemotherapy) due to inflammatory joint disease, inflammatory bowel disease, multiple sclerosis, organ transplantation, or cancer.

The Fitzpatrick skin type was type I in 115 patients, type II in 202 patients, and type III in 93 patients. The hair colour was blonde in 180 patients, brown in 203 patients, red in 20 patients, and black in 6 patients. The eye colour was blue in 177 patients, brown in 103 patients, grey in 60 patients, and green in 68 patients.

A positive family history of melanoma was reported by 46 patients.

### 3.1. Excisions

This study recorded and assessed 335 skin lesions. DD was performed in 329 cases and RCM in 105.

In 305 cases, a dermatologist performed excision based on outpatient care or imaging consultation. At the time of evaluation, 33 cases were excluded from the analysis as their histological findings were unavailable due to data protection issues or the operating doctor could not be reached.

Excisions were performed on 23 melanomas (9 invasive and 14 in situ) and 187 melanocytic nevi, including 139 dysplastic nevi, 24 basal cell carcinomas (BCCs), 3 squamous cell carcinomas (SCCs), 5 in situ SCCs, 6 melanoma metastases, 2 scars, 4 cysts, 6 seborrheic keratosis, 3 histiocytomas, 3 angiolipomas, 1 fibroma, 1 lichen planus-like keratosis, 1 angioma, 1 lipoma, 1 melanosis vulvae, and 1 lentigo solaris. A total of 271 full excisions and 1 biopsy were performed. The biopsy diagnosed a dysplastic nevus.

### 3.2. Excisions Based on Imaging Consultation and Outpatient Care

In total, 61 excisions were performed based on imaging consultation, 195 based on outpatient care (Table 2), and 16 at the patient’s request. The sixteen skin lesions excised at the patient’s request, eight melanocytic nevi and eight other benign lesions, were excluded from the analysis.

Of the 256 excisions primarily initiated by a doctor, 23 were diagnosed histologically as melanomas, with the rate based on imaging consultation (16/61, 26.2%) being significantly higher than the rate based on outpatient care (7/195, 3.6%; *p* < 0.001). Invasive (8 vs. 1; *p* < 0.001) and in situ (8 vs. 6; *p* = 0.006) melanomas were excised more often based on imaging consultation than outpatient care. The nine invasive melanomas had a tumour thickness of 0.3–0.9 mm and comprised eight at stage IA and one at stage IB. The one invasive melanoma found in outpatient care had a tumour thickness of 0.7 mm. The rate of excised melanocytic nevi based on imaging consultation was significantly lower than that based on outpatient care (23/61 [37.7%] vs. 156/195 [83.6%]; *p* < 0.001). Similar proportions of dysplastic nevi were detected based on imaging consultation and outpatient care (17/23 [73.9%] vs. 120/156 [76.9%]; *p* = 0.621). However, measured against all excisions, fewer dysplastic nevi were excised based on imaging consultation than outpatient care (17/61 [27.9%] vs. 120/195 [61.5%]; *p* < 0.001). Significantly more BCC lesions were excised based on imaging consultation than outpatient care (11/61 [18.0%] vs. 13/181 [6.7%]; *p* = 0.012).

Twenty-two non-pigmented skin lesions were excised based on imaging consultation (eleven BCC, one SCC, one in situ SCC, six melanoma metastases, two scars, and one cyst). Non-melanocytic lesions other than BCC were not compared due to insufficient numbers of cases.

The number needed to excise (NNE) was calculated for the 39 melanocytic lesions excised based on imaging consultation and the 163 melanocytic lesions excised based on outpatient care. The NNE values for melanoma (invasive and in situ; 2.4 vs. 23.3) and invasive melanoma (4.9 vs. 163.0) were smaller in imaging consultation than in outpatient care.

In imaging consultation, 3 of the 16 melanomas were found during the initial presentation. The other 13 melanomas were found after 2–6 (average 3.0) follow-up visits.

Of the seven melanomas (one invasive and six in situ) excised in the outpatient care group, two were discovered after the first imaging consultation visit and underwent no follow-up examination. We noted retrospectively that two melanomas showed a slight increase in size in the 3D-TBP. This was not noted in 600 and 1200 recorded skin lesions in these two patients. Moreover, 3D-TBP detected no change in two other melanomas during follow-up (eight months and two years). One melanoma on the ear was not recorded due to artefacts in the 3D-TBP image.

### 3.3. Digital Dermoscopy

Analog dermoscopy was used during every imaging consultation. We took 329 DD images to monitor 166 dermoscopically unclear skin lesions (1–5 follow-up examinations per lesion).

Excision was performed in 61 cases (27 based on imaging consultation and 34 based on outpatient care). Among the 27, histology confirmed 10 melanomas (6 invasive and 4 in situ), 16 nevi (including 13 dysplastic nevi), and 1 BCC. Among the 34, histology found no melanomas; it confirmed 30 nevi (including 25 dysplastic nevi), 1 BCC, and 3 in situ SCC.

Of the 26 pigmented lesions excised based on imaging consultation and examined by DD, 10 were confirmed histologically as melanomas. The NNE for DD was 2.6.

### 3.4. Reflectance Confocal Microscopy

We performed 105 RCM examinations and excised 28 skin lesions based on imaging consultation. Histology diagnosed 12 melanomas (6 invasive and 6 in situ) and 16 nevi (including 13 dysplastic nevi). The NNE for RCM was 2.3. RCM detected four melanomas not previously found by DD. Twenty-one lesions were excised during outpatient care. Histology detected no melanomas; conversely, 18 nevi (including 15 dysplastic nevi) and 3 in situ SCC were diagnosed.

### 3.5. Three-Dimensional Total Body Photography

Additionally, 3D-TBP was performed for all patients and used for monitoring over time. Five skin lesions were excised primarily due to changes in 3D-TBP. Histology diagnosed two dysplastic nevi, two invasive melanomas, and one in situ melanoma. The changes in the 3D-TBP images included size progression and pigmentation variations. 

## 4. Discussion

The combined use of TBP and DD can improve the early detection of melanoma [18,25,26]. RCM can help diagnose dermoscopically equivocal findings [27,28,29,30] and, like TBP and DD, is recommended by the European interdisciplinary guideline for melanoma in high-risk melanoma patients [2]. In addition to skin examinations of high-risk melanoma patients in outpatient care, this study used 3D-TBP, DD, and RCM during specialised imaging consultations. Excisions were performed independently in each approach, and their histological results were compared.

Significantly fewer excisions were performed, and more invasive and in situ melanomas were excised based on imaging consultation than outpatient care. The NNE for melanoma excisions was approximately ten times higher based on outpatient care than imaging consultation (23.3 vs. 2.4). The 16 melanomas (including 8 in situ and 7 invasive melanomas in stage IA and 1 invasive melanoma in stage IB) excised based on imaging consultation were diagnosed in early stages. This is consistent with the results of earlier studies in which TBP and DD primarily enabled the diagnosis of melanomas with a smaller Breslow index [17,31,32].

Most melanomas were found through imaging consultation during follow-up. DD and 3D-TBP are intended for follow-up purposes, explaining this higher diagnosis rate for melanoma [31]. DD was used to assess the progression of unclear skin lesions that did not fulfil the criteria for a malignant lesion, changed over time, or were new in 3D-TBP assessments. One-sixth of the skin lesions monitored by DD were excised based on imaging consultation, and ten melanomas were found, equalling an NNE of 2.3. The remaining lesions, which remained inconspicuous throughout follow-up, would have probably been excised if DD had been unavailable. The NNE was lower than the 5.9 stated in the literature for pigmented lesion specialists detected using dermoscopy [33]. Menzies et al. also reported a low benign-to-melanomatous skin cancer ratio of 3.5:1 using digital short-term dermoscopy, demonstrating the benefits of DD [23]. Notably, a sixth of the skin lesions deemed benign in imaging consultation using DD but excised in outpatient care showed no histological evidence of melanoma. Therefore, DD led to fewer excisions of unclear lesions.

RCM was primarily used for equivocal dermoscopic findings. A quarter of the lesions examined by RCM were excised, and 12 melanomas were histologically confirmed, corresponding to an NNE of 2.3, similar to the NNE reported in the literature [30]. Four of these melanomas had previously been classified by DD as benign nevi and would probably not have been excised had RCM not been performed. Therefore, adding RCM was advantageous over the TBP and DD two-step programme. Notably, a quarter of the skin lesions deemed benign using RCM in the imaging consultation were excised in outpatient care but showed no histological evidence of melanoma. Due to availability, the Vivascope 3000 handheld device was used for RCM in this study. With this device, only a small section of 750 μm × 750 μm can be viewed, in contrast to the Vivascope 1500, which records a mosaic over 8 × 8 mm. With the handheld device, there is a risk of missing relevant parts of a lesion, which is why the Vivascope 1500 should be used for pigmented lesions, in particular, if available.

In addition, 3D-TBP was performed on every visit and considered during the skin lesion assessments. A conspicuous progressive lesion size increase or pigment change led to excision in five cases, of which three were histologically confirmed as melanomas. The automatic detection and sequential comparison of lesions made available by the three-dimensional variant of TBP were crucial to identifying these changes in some of the many nevi. Limitations of 3D-TBP include its limited ability to assess the skin under the hair, on the foot soles, and in skin folds (e.g., rima ani, submammary fold, and abdominal fold) [34], as well as the reduced resolution of zoomed-in skin lesions, the lack of depth in the information on slightly raised lesions, and unfavourable light reflections.

By combining 3D-TBP, DD, and RCM into a three-step procedure, the number of excisions based on imaging consultation was evidently lower than based on outpatient care. In addition to its more precise diagnosis, the three-step procedure enabled the monitoring of lesions with unclear changes during follow-up. Such suspicious skin lesions would likely have been excised in outpatient care had these follow-up imaging options been unavailable.

Images generated by 3D-TBP, DD and RCM are suitable for teledermatologic assessment. Several studies have shown that teledermatology can reduce waiting times for initial assessment [35] while being highly effective [36,37]. Artificial intelligence showed the potential to improve diagnostic accuracy for these devices even more [38]. Teledermatology could be particularly helpful for the general population who do not have the opportunity to visit a referral centre.

## 5. Limitations

This study used a three-step procedure to assess pigmented skin lesions. Even though DD was only used for lesions deemed unclear during the skin and 3D-TBP examinations, and RCM was only used when dermoscopy findings were unclear, the excision decision was made based on all findings. Therefore, the calculated NNE for the individual diagnostic methods was hypothetical and possibly lower than if the findings had been assessed separately.

In Germany, it is common to carry out skin examinations in high-risk melanoma patients alternately in outpatient care and specialised centres. The follow-up examinations in outpatient care are carried out quarterly, semi-annually, or once a year. However, the imaging consultation and outpatient care follow-up visits were not always synchronised, limiting comparability.

The histological findings were obtained by different pathologists in different laboratories. The histological findings of different pathologists may differ, which reduces comparability. However, findings and images from the imaging consultation were not supplied to the pathologists.

## 6. Conclusions

This study compared findings of skin examinations through specialised imaging consultation using 3D-TBP, DD, and RCM and outpatient care where dermoscopy was performed as standard care in the same high-risk patient cohort. This three-step method achieved a ten-times-lower NNE for melanoma excisions with fewer excisions overall. This achievement was due to the accurate diagnosis made by RCM and possibly the precise follow-up with DD and 3D-TBP that replaced diagnostic excisions. No melanoma was detected histologically in lesions deemed benign based on imaging consultation but excised in outpatient care, which underlines the reliability of the imaging assessment. RCM is a useful addition to skin examinations, with 3D-TBP and DD largely used for equivocal dermoscopic findings.

## Figures and Tables

**Table 1:** Patient characteristics, follow-up visits, and existing risk factors for developing melanoma at study recruitment.

Patient Characteristics	
Patients (*n*)	410
Male (*n*)	224 (54.6%)
Female (*n*)	186 (45.4%)
Age (mean)	18–84 (mean 51.6, SD 13.1) years
Follow-up visits (*n*)	1070
Follow-ups per patient (*n*)	1–8 (mean 2.1, SD 1.1)
Follow-up period	0–33 (mean 14.9, SD 8.5) months
Time between visits	1–24 (mean 8.4, SD 3.4) months
Skin type (Fitzpatrick)	
Skin type I (*n*)	115 (28.0%)
Skin type II (*n*)	202 (49.3%)
Skin type III (*n*)	93 (22.7%)
Hair colour	
Blond (*n*)	180 (43,9%)
Brown (*n*)	203 (49.5)
Red (*n*)	20 (4.9%)
Black (*n*)	6 (1.5%)
Missing (*n*)	1 (0.2%)
Eye colour	
Blue (*n*)	177 (43.2%)
Brown (*n*)	103 (25.1%)
Grey (*n*)	60 (14.6%)
Green (*n*)	68 (16.6%)
Missing (*n*)	2 (0.5%)
Risk factors	
Nevi count	
<50 melanocytic nevi (*n*)	36 (8.8%)
>50 melanocytic nevi (*n*)	70 (17.1%)
>100 melanocytic nevi (*n*)	304 (74.1%)
Missing (*n*)	1 (0.3%)
Dysplastic nevi (*n*)	257 (62.7%)
Genetic risk for melanoma (*n*)	7 (1.7%)
Immunosuppression (*n*)	16 (3.9%)
Positive family history (*n*)	46 (11.2%)

**Table 2:** Comparison of histological findings of excisions initiated within the imaging consultation and in outpatient care (NA: not applicable).

	Imaging Consultation, *n* (%)	Outpatient Care, *n* (%)	
Number of excisions	61	195	
Melanoma	16 (26.2%)	7 (3.6%)	*p* < 0.001
Invasive melanoma	8 (13.1%)	1 (0.5%)	*p* < 0.001
In situ melanoma	8 (13.1%)	6 (3.1%)	*p* < 0.001
All nevi	23 (37.7%)	156 (83.6%)	*p* < 0.001
Dysplastic nevi	17 (27.9%)	120 (61.5%)	*p* < 0.001
Other lesions	22 (36.1%)	32 (16.4%)	NA
Basal cell carcinoma	11 (18.0%)	13 (6.7%)	*p* = 0.012
Squamous cell carcinoma	1 (1.6%)	2 (1.0%)	*p* = 0.560
Actinic keratosis	1 (1.6%)	4 (2.1%)	*p* = 1.000
Melanoma metastasis	6 (9.8%)	0 (0%)	NA
Scar	2 (3.3%)	0 (0%)	NA
Cyst	1 (1.6%)	3 (1.5%)	NA
Seborrheic keratosis	0 (0%)	5 (2.6%)	NA
Histiocytoma	0 (0%)	1 (0.5%)	NA
Fibroma	0 (0%)	1 (0.5%)	NA
Lichen planus-like keratosis	0 (0%)	1 (0.5%)	NA
Melanosis vulvae	0 (0%)	1 (0.5%)	NA
Lentigo solaris	0 (0%)	1 (0.5%)	NA

## Data Availability

The data that support the findings of this study are available on request from the corresponding author. The data are not publicly available due to privacy or ethical restrictions. Further inquiries can be directed to the corresponding author.

## Abbreviations

TBP	total body photography
3D-TBP	three-dimensional total body photography
DD	digital dermoscopy
RCM	reflectance confocal microscopy
BCC	basal cell carcinoma
SCC	squamous cell carcinoma
SD	standard deviation
NNE	number needed to excise
